# Nanog Predicts Poor Prognosis in Human Pancreatic Cancer and Is Downregulated by QingyihuaJi Formula in Pancreatic Cancer Stem Cells

**DOI:** 10.1155/2016/7028289

**Published:** 2016-10-17

**Authors:** Song Gao, Yan Pan, Libin Song, Lei Dong, Lao I. Weng, Peng Wang, Yongqiang Hua, Zhen Chen, Luming Liu

**Affiliations:** ^1^Department of Integrative Oncology, Fudan University Shanghai Cancer Center, Shanghai 200032, China; ^2^Department of Oncology, Shanghai Medical College, Fudan University, Shanghai 200032, China; ^3^Department of Pathology, Fudan University Shanghai Cancer Center, Shanghai 200032, China

## Abstract

Qingyihuaji formula (QYHJ), confirmed efficacious in a series of clinical trials, has been applied to human pancreatic carcinoma treatment in Shanghai Cancer Center for years. Recent evidence highlighted that pluripotent stem cells transcription factor Nanog plays a pivotal role in carcinogenesis. However, there is little published information regarding the underlying clinical significance and mechanisms of transcription factor Nanog in pancreatic cancer. In this study, our results indicated that Nanog is overexpressed in human pancreatic cancer stem cells and downregulated by QYHJ, which may contribute to explain the clinical effectiveness of QYHJ and provide advanced pancreatic cancer patients with a new therapeutic option, supporting our hypothesis that the degradation pathway is another mechanism by which QYHJ affects Nanog expression.

## 1. Introduction

Pancreatic cancer is a devastating neoplasm and ranks the fourth among the most common causes of cancer deaths worldwide [1]. Although great advances have been made in the treatment of pancreatic cancer, the conventional therapies, such as surgery, radiotherapy, and chemotherapy, are limited and the prognosis is dismal [2, 3]. Therefore, the median overall survival is only 5-6 months after conventional therapies for locally advanced and metastatic disease. Consequently, the five-year survival rate of PDAC is merely 6% [4]. Thus, a better understanding of the genetic alterations and detailed molecular mechanisms involved in pancreatic cancer development will facilitate advances in the prevention, diagnosis, and treatment of this lethal disease.

Qingyihuaji formula (QYHJ), composed of traditional Chinese herbs, has been applied to human pancreatic cancer treatment for many years. Our clinical study indicates that the treatment with QYHJ combined with chemotherapy has prolonged survival time of advanced pancreatic cancer patients [5, 6]. However, its underlying mechanism remains to be elucidated.

The transcription factor Nanog controls stemness acting as a key determinant of both embryonic stem cells self-renewal and differentiated somatic cells reprogramming to pluripotency. Nanog is a member of ANTP class NK family genes and plays a key role in stem cell self-renewal and pluripotency differentiation [7]. In addition to self-renewal regulation of embryonic development, the abnormal expression of Nanog gene is found in malignant germ cell tumors, such as embryonic carcinoma and seminoma [8]. The abnormal expression of Nanog is also detected in solid tumors, such as breast [9], gastrointestinal [10], and kidney [11] cancer. Here, we found that QYHJ treatment could downregulate the expression of Nanog. We assume that the expression of Nanog may be coordinated and downregulated by QYHJ* in vitro *and* in vivo*. Since aberrant Nanog expression occurs in tumorigenesis, therapeutics targeted Nanog may improve the outcomes of patients with pancreatic cancer, thus suggesting the use of QYHJ to further improve preventive and therapeutic approaches in patients with this devastating disease.

Thus, the aim of the study is to evaluate Nanog expression in human pancreatic cancer tissue and the effects of QYHJ on transcription factor Nanog in human pancreatic cancer stem cell.

## 2. Materials and Methods

### 2.1. Cell Lines and Reagents

Pancreatic cancer cell line PANC-1 was obtained from the Shanghai Cell Bank of the Chinese Academy of Sciences (Shanghai, China) and grown in complete growth medium as recommended by the manufacturer. CD24+/CD44+/ESA+ subpopulation cells were isolated from the pancreatic cancer cell lines PANC-1 as the previous description [12]. The cultured cells were maintained in a humidified 5% CO2 atmosphere at 37°C.

### 2.2. Drugs and Reagents

QYHJ formula, comprised of seven Chinese herb medicines (Hedyotidis herba,* Amorphophallus konjac*, herba scutellariae barbatae,* Coix* seed, akebia stem, fiveleaf gynostemma herb, and java amomum fruit), was prepared as previously described [13–15]. In short, QYHJ powder was obtained from Jiangyin Tianjiang Pharmaceutical Co., Ltd. To ensure standardization and maintain the interbatch reliability of QYHJ, a high performance liquid chromatography (HPLC) chromatographic fingerprint was developed for quality control. The fingerprint chromatograms of QYHJ formula are shown in our previous report [13–15]. The final decoction of QYHJ was prepared after dissolving the herbal powder in distilled water to the required concentration. The daily dosage for nude mice was 36 g/kg, once per day p.o., respectively, calculated according to the following human-mouse transfer formula: *D*_*b*_ = *D*_*a*_ × (*R*_*b*_/*R*_*a*_)×(*W*_*b*_/*W*_*a*_)2/3, where *D*, *R*, and *W* represent dosage, shape coefficient, and body weight, respectively, and *a* and *b* represent human and mouse, respectively. Gemcitabine (Eli Lilly) was dissolved in sterile PBS, 25 mg/kg [14, 15], twice per week by intraperitoneal injection. Antibodies of rabbit anti-human Nanog were purchased from Abcam. CCK-8 were purchased from Dojindo Molecular Technologies, Inc., and BD Matrigel™ Matrix basement membrane was purchased from BD Biosciences. Transwell insert (8 *μ*m) was purchased from Corning Incorporated.

### 2.3. Cell Proliferation Assays

A total of 5 × 10^3^ cells were plated in duplicate wells of 96-well plates and allowed to adhere overnight. Cells were then treated with human recombinant QYHJ at concentrations ranging from 0 to 200 mg/mL. After 48 hours, indices of cell proliferation were determined with Cell Counting Kit-8 (Dojindo Molecular Technologies, Inc., Gaithersburg, MD).

### 2.4. Cell Cycle Analysis

For cell cycle analysis of cells, cells growing at low density (40%) in normal growth medium were treated with QYHJ (32 mg/mL) or vehicle. After 24 h, cells were fixed with 1% paraformaldehyde in PBS (phosphate-buffered saline) for 15 minutes and refixed with 70% ethanol. The cells were then treated following the standardized protocol, and cell cycle analyses were done by flow cytometry as described previously [12].

### 2.5. Cell Invasion and Migration Assays

Transwell chambers were coated with Matrigel for 3 h to form a basement membrane prior to use. Following treatment with QYHJ (32 mg/mL) for 24 h, the CD24+/CD44+/ESA+ PCSCs were harvested and seeded in the upper chamber at a density of 30,000 cells/well in 200 *μ*L free-serum culture medium. Culture medium containing 10% FBS (600 *μ*L) was added in the lower chamber as a chemoattractant. After incubation for 24 h at 37°C, all of the noninvaded cells were removed from the upper face of the membrane with a cotton swab. The invaded cells on the lower face of membrane were then fixed for 10 min with 4% paraformaldehyde (Genmed Scientifics, Inc., Arlington, MA, USA) and stained with 0.2% crystal violet for 15 min. Cells that had invaded through the membrane were counted using the inverted microscope. The general procedure of the migration assay was similar to that of the invasion assay, with the exception that the membranes in each chamber were not coated with Matrigel and the number of cells seeded in the upper chamber was 5 × 10^4^ cells/well.

### 2.6. Colony Formation Assay

For colony formation, CD24+/CD44+/ESA+ PCSCs were seeded (500 cells/well) in six-well plates overnight. After 14 days, the culture medium was removed, and cells were briefly rinsed with PBS. The cells were then fixed with 4% paraformaldehyde and stained with 0.1% crystal violet, and colonies were counted by visual inspection.

### 2.7. RNA Extraction and Quantitative Real-Time PCR (qRT-PCR)

Total RNA was extracted from frozen plasma or cultured cell lines using TRIzol reagent (Invitrogen, USA) according to the manufacturer's instructions. With GAPDH as an internal control, qRT-PCR was performed for Nanog. Total RNA was then converted to cDNA by reverse transcription using oligo-dT primers and SuperScript II reverse transcriptase (Invitrogen). For qRT-PCR, three replicates of each sample were amplified in a 20 *μ*L reaction mixture containing SYBR Green reaction mix (Qiagen, Germany) and 0.5 mM of primer and analyzed using a Roche Light-Cycler (Roche, Basel, Switzerland). The relative gene expression in cells was determined using the comparative delta-delta CT method (2^−ΔΔCt^) and the fold change in gene expression of tissues was calculated using the standard ΔΔCT method. The primer sequences of Nanog are sense: 5′-GCAATGGTGTGACGCAGAAGGC-3′, antisense: 5′-TGGGTCTGGTTGCTCCAGGTTG-3′; GAPDH sense: 5′-CAAGGTCATCCATGACAACTTTG-3′, antisense: 5′-GTCCACCACCCTGTTGCTGTAG-3′.

### 2.8. Western Blot Analysis

CD24+/CD44+/ESA+ PCSCs were washed in PBS and lysed with RIPA buffer (Invitrogen), and a bicinchoninic acid protein assay kit (Pierce, Rockford, IL, USA) was used to calculate the protein concentration of each sample. Equivalent amounts of proteins were separated by SDS-PAGE and transferred to polyvinylidene fluoride membranes for immunoblotting. The membranes were blocked in 5% fat-free milk for 2 hours at room temperature, washed three times, then incubated with the following primary antibodies: rabbit anti-human Nanog antibody (1 : 200, lot : GR154878-3, Santa Cruz Biotechnology, USA) and rabbit anti-human GAPDH antibody (1 : 1000, #ab18162, Abcam). GAPDH was used as a loading control. Horseradish peroxidase conjugated secondary antibodies (Cell Signaling Technology, Boston, USA) and an ECL chemiluminescence kit were used to detect bound antibody. Western blot was performed according to standard protocols [16].

### 2.9. *In Vivo* Xenograft Experiments

Female BALB/c-nu/nu nude mice, 4 to 6 weeks old, were obtained from Shanghai Laboratory Animal Center, Chinese Academy of Sciences (Shanghai, China), and housed in laminar flow cabinets under specific pathogen-free conditions with food and water* ad libitum*. The study protocol was approved by the Shanghai Medical Experimental Animal Care Committee. For QYHJ treatment, an appropriate concentration (36 g/kg) of QYHJ and gemcitabine 25 mg/kg, twice per week by i.p. injection, were fed to nude mice.

### 2.10. Immunohistochemical Analysis

Paraffin-embedded sections (4 *μ*m) were stained with primary antibodies for Nanog (1 : 200) and then stained with HRP conjugated secondary antibodies. Sections were developed in diaminobenzidine and counterstained with hematoxylin. Images were taken, and five fields (200x) selected randomly from each section were examined. For the data quantification, integrated optical density (IOD) of positive expression was measured by Image Pro Plus 6.0 software.

### 2.11. Statistical Analysis

Kaplan-Meier analysis was used to evaluate OS, and log-rank test was used to compare OS between groups. The data were expressed as mean ± standard deviation. ANOVA and Student's *t*-test were used to determine the statistical significance of differences between experimental groups, with *p* < 0.05 as the statistically significant level.

## 3. Results

### 3.1. Nanog Overexpression Is Associated with Clinicopathological Characteristics and Prognosis of Pancreatic Cancer Patients

**Figure 1:**
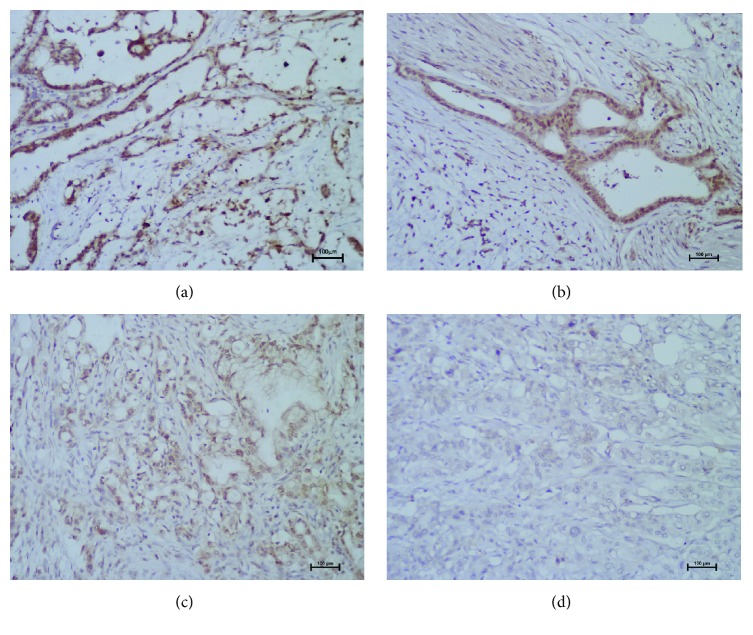
Immunohistochemical analysis of Nanog in PDAC (pancreatic duct adenocarcinoma) tissues. In PDAC tissues, immunoreactivity for Nanog was observed in the nucleus or nucleoplasm of cancer cells (a–c), with no immunoreactivity in the surrounding stroma (d). The immunoreactivity was different in the respective cases: (a) strong; (b) moderate; (c) weak; and (d) absent expression (scale bar, 100 *μ*m).

**Figure 2:**
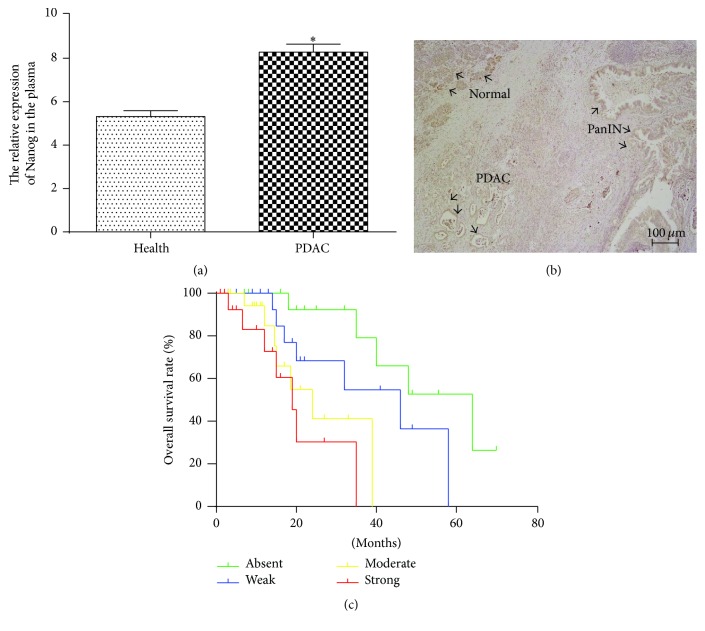
Nanog is aberrantly overexpressed in plasma and tissues and associated prognosis of patients with pancreatic cancer. *∗* indicates *p* value < 0.05.

To investigate the clinical relevance of the transcription factor Nanog in pancreatic cancer, we measured the expression levels of Nanog protein in 47 paraffin-embedded human PDAC samples by immunohistochemistry. As described in Section 2, the expression of Nanog was evaluated in terms of intensity and percentage separately and finally expressed as strong, moderate, weak, or absent. The representative images are shown in Figure 1 and the IHC photo of Nanog from PanIN to PDAC was shown in Figure 2(b). We also analyzed the relationship between the intensity of Nanog staining and clinicopathological features. Statistical analysis confirmed that Nanog overexpression was correlated with tumor size, TNM stage, and liver metastasis (Table 1). Furthermore, survival analysis displayed that higher Nanog staining intensity correlated with poorer prognosis in pancreatic cancer patients (log-rank test, *p* < 0.001, Figure 2(c)). Moreover, the univariate analysis revealed that large tumor size, advanced TNM stage, liver metastasis, and high Nanog expression were significantly associated with an increased risk of cancer-related death. The multivariate analysis demonstrated that, of note, high expression levels of Nanog were found to be significantly associated with poorer survival in patients (*p* < 0.001, Table 2). Meanwhile, the levels of Nanog in the plasma were detected between pancreatic cancer patients and health peoples, we found that lower plasma Nanog levels were significantly higher in patients with advanced pancreatic cancer, compared to the healthy group (Figure 2(a)).

### 3.2. QYHJ Treatment Can Reduce the Tumorigenicity of CD24+/CD44+/ESA+ PCSCs* In Vivo*

**Figure 3:**
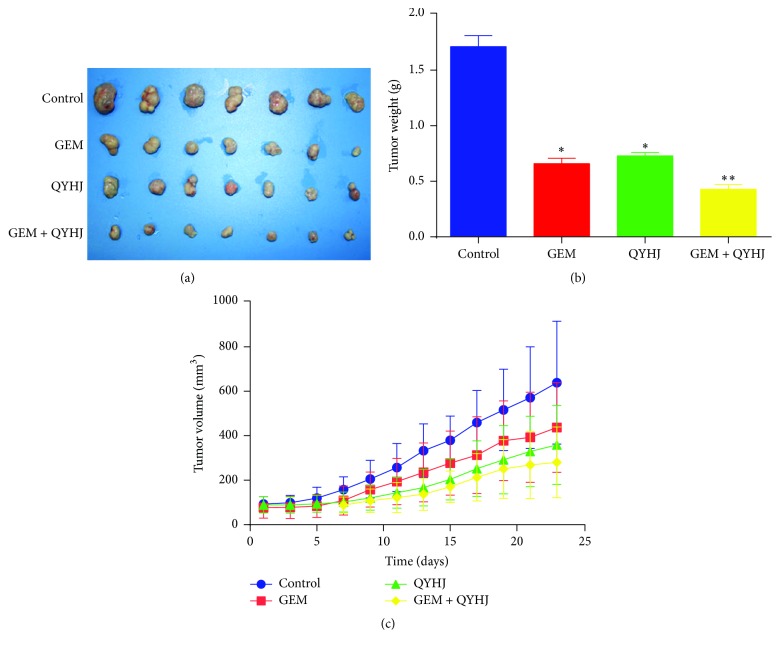
QYHJ treatment can reduce the tumorigenicity of PCSCs in nude mice. *∗* indicates *p* value < 0.05 and *∗∗* indicates *p* value < 0.01.

To examine the effect of the inhibition of QYHJ on the tumorigenic ability of PCSCs* in vivo*, CD24+/CD44+/ESA+ PCSCs were inoculated subcutaneously into nude mice. Both groups of nude mice were fed under the same conditions. Two weeks after injection, nude mice treated with saline had an obvious swelling at the injection site, whereas the nude mice treated with QYHJ did not demonstrate any swelling. 23 days after injection, all of the mice were sacrificed. Although the mice in both groups had varying sizes of subcutaneous tumors on their back, the results showed that subcutaneous tumors treated with QYHJ were significantly smaller in both size (*p* < 0.01, Figure 3(c)) and weight (*p* < 0.01, Figure 3(b)) than control group, which is treated with saline.

### 3.3. QYHJ Downregulates Nanog Expression in Tumor Samples

**Figure 4:**
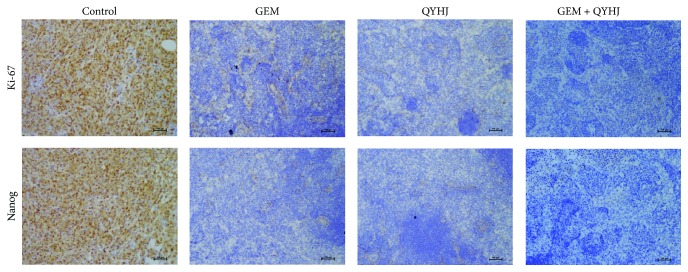
Immunohistochemistry analyses on expression of Nanog/Ki-67 after QYHJ and GEM treatment in pancreatic tumor tissues (scale bar, 100 *μ*m).

Although QYHJ effectiveness has been confirmed in a series of clinical trials [5, 6], the mechanisms by which it exerts its effects are not completely understood. To explore the changes in the protein levels of Nanog following treatment with QYHJ, we conducted immunohistochemical staining in tumor samples. Our results showed that the expression of Nanog and Ki-67 protein in the tumor tissues treated with QYHJ and GEM was reduced, whereas their expression in tumor tissues in control group was only weakly reduced or even enhanced (Figure 4). In summary, these experiments* in vivo* show that when PCSCs were treated with QYHJ and GEM, the tumorigenicity of these cells in nude mice is reduced and the expression of Nanog and Ki-67 is also downregulated. In summary, our results also showed that cotreatment with QYHJ and GEM significantly inhibited the expression of Nanog at protein level* in vivo*.

### 3.4. QYHJ Treatment Results in the Inhibition of* In Vitro *Proliferation and Invasion of PCSCs

**Figure 5:**
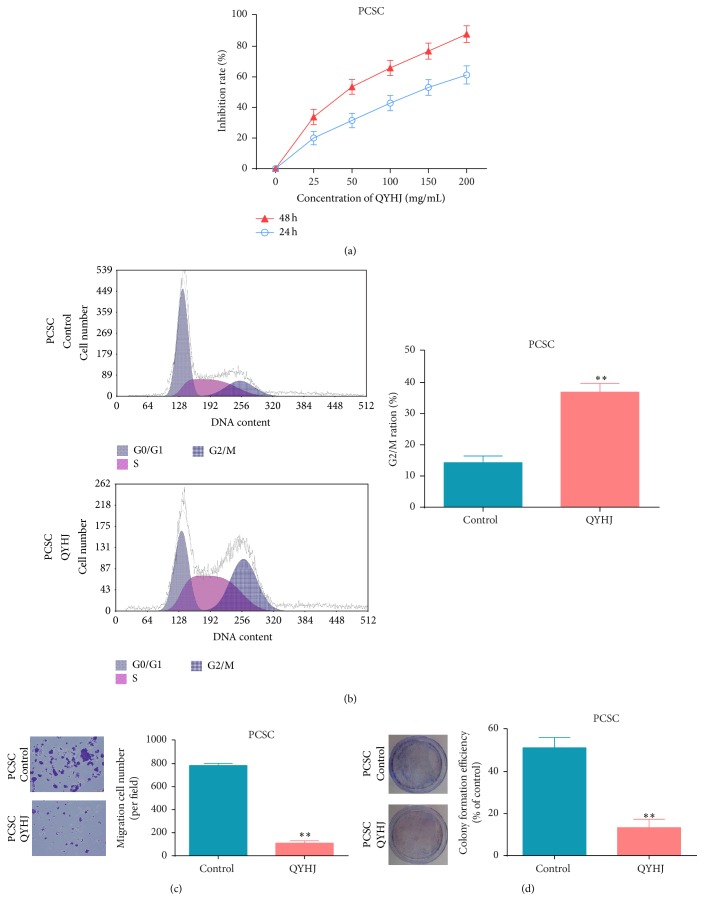
QYHJ treatment can effectively inhibit the* in vitro* proliferation, invasion, and cell cycle progression of pancreatic cancer stem cells. *∗∗* indicates *p* value < 0.01.

Using a cell proliferation assay, we observed that the* in vitro* proliferation rate of PCSCs (from the PANC-1 cell lines) treated with QYHJ was significantly lower than control group (*p* < 0.01, Figure 5(a)). Next, we analyzed the proportion of cells in various stages of the cell cycle using flow cytometry, and the results indicate that QYHJ results in the blockage of PCSCs at the G2/M phase in the cell cycle and inhibits their proliferation (*p* < 0.01, Figure 5(b)). Further, the transwell invasion assay results showed that the number of cells invaded under the membrane in PCSCs treated with QYHJ was significantly less than those transfected with control (*p* < 0.01, Figure 5(d)).

At the same time, the soft agar colony formation assay results showed that the colony formation rate of PCSCs treated with QYHJ in soft agar was significantly lower than control group (*p* < 0.01, Figure 5(c)). These experiments show that treatment with QYHJ can effectively inhibit the* in vitro *proliferation and invasive ability of PCSCs.

## 4. Discussion

Due to the poor prognosis, there is an urgent need to prolong pancreatic cancer patients' lifetime. Previous studies have shown only limited benefit with current treatments. The prognosis of PDAC is extremely dismal and the disease is obviously resistant to current therapies [16].

Traditional Chinese medicine has had an advantage in cancer therapy for thousands of years. QYHJ has shown promising outcomes in treatment for patients with pancreatic cancer. In QYHJ treatment group, the survival rate of 5 years was 8.4%, and the median survival time was 7.6 months, obviously longer than that of control group [4]. QYHJ could benefit advanced pancreatic cancer patients by stabilizing tumors and prolonging life expectancy, with fewer side effects than those of chemotherapy or radiotherapy [5]. Another retrospective study also showed that, from January 2002 to December 2007, patients who took QYHJ based integrative treatment have much longer survival, 20 cases survive more than 3 years, of which 13 cases survive more than 5 years, and the longest survival time is 169 months [6]. These studies indicated that QYHJ might be a novel therapeutic option for pancreatic cancer treatment. Therefore, it is valuable to explore its antitumor mechanism.

Little is known about the clinical and biological function of transcription factor Nanog thus far. To our knowledge, the findings of our current study provide the first evidence of the potential clinical utility of Nanog expression as a prognostic factor in pancreatic carcinoma. Our current works indicate that Nanog overexpression correlates with TNM stage and prognosis of patients with pancreatic cancer. Strong evidences have suggested that overexpression of Nanog is closely related to tumorigenesis, tumor metastasis, and distant recurrence after chemoradiotherapy [17, 18]. Nanog was found to be upregulated, which enhanced the expression of typical cancer stem cell markers, such as CD44, CD133, and ALDH1A1 in prostate cancer [19]. However, the role of transcription factor Nanog in pancreatic cancer stem cells is still elusive.

In a previous study, we demonstrated that QYHJ exerted an inhibitory effect on the growth of human pancreatic cancer cell and liver metastasis, perhaps by targeting VEGF and Cyr61 [20]. However, the mechanisms leading to growth arrest remain unclear. In the current study, we observed that QYHJ downregulated Nanog expression in cancer stem cells at a transcriptional level as well as promoted degradation. We also evaluated Nanog protein expression in human pancreatic cancer tissue by immunohistochemistry. We found that Nanog is upregulated in human pancreatic cancer tissues compared to normal adjacent pancreatic tissues. These results indicate that overexpression of Nanog may play a key role in the development or the progression of pancreatic cancer.

In conclusion, we found that Nanog is overexpressed in pancreatic cancer tissues and its expression is downregulated* in vitro *and* in vivo *by QYHJ. QYHJ inhibited the proliferation and invasion of human pancreatic cancer stem cell PANC-1, partly through downregulation of transcription factor Nanog expression. Nanog acts as a therapeutic target of QYHJ in the treatment of pancreatic cancer stem cells, and its expression status mediates different responses to QYHJ treatment of pancreatic cancer stem cells.

## Figures and Tables

**Table 1:** Correlation of Nanog expression and clinicopathological characteristics in patients with pancreatic cancer.

Parameters		Nanog IHC scores (*N* = 47)				*p* value
		3 (*N* = 12)	2 (*N* = 15)	1 (*N* = 11)	0 (*N* = 9)	
*Age*						
Mean (Range)	57.5 (35~78)	58.1 (52~76)	56.9 (47~78)	51.2 (39~75)	53.3 (35~71)	0.133^**#**^
*Gender*						
Male	26	8	7	6	5	
Female	21	4	8	5	4	0.491
*Tumor range*						
Confined	26	5	12	7	2	
Invasion of adjacent organ	15	6	2	3	4	
Distal metastasis	6	3	1	0	2	0.475
*Tumor size*						
≤4 cm	19	13	4	1	1	
>4 cm	28	12	8	5	3	0.041^**∗**^
*Tumor location*						
Head	34	18	7	8	2	
Body and tail	13	6	4	2	1	0.832
*TNM stage*						
I and II	38	14	7	9	9	
III and IV	9	5	3	1	0	<0.001^**∗**^
*Differentiation*						
Well and moderate	35	12	5	11	7	
Poor	12	8	2	1	1	0.012
*Liver metastasis*						
Yes	24	8	9	4	3	
No	23	5	4	5	9	0.037^**∗**^
*CA19-9 (IU/mL)*						
≤500	9	2	3	1	3	
>500	36					0.067

All the other *p *values were calculated by Pearson Chi-square test. #: spearman rank correlation test. *∗* denotes statistical significance.

**Table 2:** Univariate and multivariate analysis of prognostic factors influencing overall survival in 47 patients with pancreatic ductal adenocarcinoma undergoing surgery.

Parameter	Univariate analysis			Multivariate analysis		
	HR	95% CI	*p* value	HR	95% CI	*p* value
*Age*	1.1	0.5–2.4	0.53			
*Gender*						
Male						
Female	0.79	0.4–1.3	0.176			
*Tumor range*						
Confined						
Invasion of adjacent organ	3.3	1.65–7.21				
Distal metastasis	4.1	2.65–10.69	0.61			
*Tumor size*						
≤4 cm						
>4 cm	2.1	1.8–4.9	0.041^*∗*^	1.3	0.57–3.77	0.19
*Tumor location*						
Head						
Body and tail	0.455	0.17–1.10	0.057			
*TNM stage*						
I and II						
III and IV	4.0	1.2–8.3	<0.001^*∗*^	8.9	3.6–53.5	0.32
*Differentiation*						
Well and moderate						
Poor	8.5	4.3–76.9	0.55			
*Liver metastasis*						
Yes						
No	5.8	1.5–14.9	0.0013^*∗*^	6.6	0.89–14.2	0.062
*Nanog expression*						
Strong						
Moderate	1.55	0.55–4.11		1.56	0.60–4.11	<0.001^*∗*^
Weak	3.0	0.4–20.1		3.0	0.77–20.5	0.0013^*∗*^
Absent	7.65	5.21–32.3	<0.001^*∗*^	8.10	4.22–25.0	<0.01^*∗*^

HR, hazard ratio; CI, confidence interval. *∗* denotes statistical significance.
